# Compromised Dynamic Cerebral Autoregulation in Patients With Central Disorders of Hypersomnolence

**DOI:** 10.3389/fneur.2021.634660

**Published:** 2021-03-11

**Authors:** Fang Deng, Yanan Zhang, Ran Zhang, Qi Tang, Zhenni Guo, Yudan Lv, Zan Wang, Yi Yang

**Affiliations:** ^1^Department of Neurology, The First Hospital of Jilin University, Changchun, China; ^2^Department of Neurology, Clinical Trial and Research Center for Stroke, The First Hospital of Jilin University, Changchun, China

**Keywords:** central disorders of hypersomnolence, narcolepsy type 1, idiopathic hypersomnia, rapid-eye-movement sleep behavior disorder, dynamic cerebral autoregulation

## Abstract

**Objective:** We aimed to investigate the dynamic cerebral autoregulation (dCA) in patients with central disorders of hypersomnolence during wakefulness.

**Methods:** Thirty-six patients with central disorders of hypersomnolence were divided into three groups according to polysomnography and multiple sleep latency test results: the idiopathic hypersomnia group (IH), narcolepsy type 1 without rapid-eye-movement sleep behavior disorder group (NT1-RBD), and narcolepsy type 1 with rapid-eye-movement sleep behavior disorder group (NT1 + RBD), with 12 patients in each group. Twelve sex- and age-matched healthy controls were recruited. We assessed the Epworth sleepiness scale (ESS) and dCA of all subjects. dCA was assessed by analyzing the phase difference (PD) using transfer function analysis. The ESS and dCA were analyzed before and after standardized treatment in 24 patients with narcolepsy type 1.

**Results:** The overall PD of the IH, NT1-RBD, and NT1 + RBD groups were lower than that of the control group (*P* < 0.001). There were no significant differences between the overall PD of the NT1-RBD and NT1 + RBD group (*P* > 0.05). The ESS scores decreased and the overall PD increased after treatment in 24 patients with narcolepsy type 1 (*P* < 0.001). Multivariable analysis showed that mean sleep latency in multiple sleep latency test was independently associated with impaired overall PD (*P* < 0.05).

**Conclusions:** The dCA is impaired in patients with central disorders of hypersomnolence. The impairment of dCA occurs irrespective of NT1-RBD/+RBD. The ESS score and dCA improved in patients with narcolepsy type 1 after medication treatment. The mean sleep latency in multiple sleep latency test was independently associated with impaired dCA.

**Clinical Trial Registration:** www.ClinicalTrials.gov, identifier: NCT02752139.

## Introduction

The central disorders of hypersomnolence include narcolepsy type 1 (NT1), narcolepsy type 2 (NT2), and idiopathic hypersomnia (IH) (1). Studies have demonstrated a decreased cerebral blood flow (CBF) in patients with NT1 and IH (2, 3), which implies that cerebral autoregulation (CA) function may play an important role in the central disorders of hypersomnolence. CA is a physiological mechanism of the brain that maintains sufficient CBF despite changes in the blood or cerebral perfusion pressure. Dynamic cerebral autoregulation (dCA) can respond to real-time changes in the blood pressure within seconds, allowing continuous measurement of CA and a wave by wave analysis of hemodynamics (4). Many diseases may impair dCA, including cerebrovascular diseases, traumatic brain injury and Alzheimer's disease (5, 6). The dCA mechanism is complex; it includes myogenic, metabolic, endothelial, and neurogenic mechanisms, and neurogenic including the monoaminergic and cholinergic mechanisms (7). Studies have found the monoaminergic mechanism including 5-hydroxytryptamine and norepinephrine to be implicated in the central disorders of hypersomnolence (8, 9), which are vasoactive substances and likely affect the dCA (10, 11).

Rapid-eye-movement sleep behavior disorder (RBD) affects 30–63% of NT1 patients (12). Idiopathic RBD exhibits decreased cerebral perfusion and dCA (13, 14); however, there are currently no studies on RBD and dCA in NT1 patients. We hypothesize that dCA is impaired in patients with central disorders of hypersomnolence. We obtained dCA data from patients with IH and NT1 and investigated the relationship between central disorders of hypersomnolence and dCA.

## Methods

The study was approved by the ethics committee of the First Hospital of Jilin University and followed the guidelines of the Declaration of Helsinki (1964). Written informed consent was obtained from all the participants or their relevant guardians.

### Subjects

Patients with central disorders of hypersomnolence, that underwent the polysomnography (PSG) and multiple sleep latency tests (MSLT), were recruited from the Department of Neurology, First Hospital of Jilin University, from March 2018 to March 2019. A total of 36 patients were enrolled with central disorders of hypersomnolence [12 patients with IH, 12 with NT1 without RBD group (NT1-RBD), and 12 with NT1 with RBD group (NT1 + RBD), respectively]. Twelve age- and sex-matched healthy controls were also recruited from the same region, based on their PSG results.

#### Inclusion Criteria

The inclusion criteria for the different groups were as follows: (1) Control group: age- and sex-matched healthy volunteers without sleep disorder in PSG; (2) NT1-RBD and NT1 + RBD groups: subjective sleepiness, cataplexy, mean sleep latency ≤8 min, and ≥2 sleep-onset rapid-eye-movement (REM) periods (SOREMP) on MSLT or one SOREMP on the preceding night PSG coupled with one SOREMP on the MSLT. The diagnostic criteria of RBD was based on the International Classification of Sleep Disorders 3rd edition; (3) IH group: subjective sleepiness, absence of cataplexy, mean sleep latency ≤8 min, and with one time or without SOREMP (including no SOREMP on the PSG from the preceding night); (4) the age cut-off value being 15 to 65; and (5) good bilateral temporal window penetration.

#### Exclusion Criteria

The exclusion criteria were as follow: presence of (1) sleep-related breathing disorders, circadian rhythm disorders, and other causes of disturbed nocturnal sleep; (2) history of secondary daytime sleepiness, epilepsy, mental illness, or drug abuse; (3) arrhythmia, hyperthyroidism, and other hemodynamic factors; (4) intracranial and extracranial vascular stenosis or occlusion diagnosed by vascular ultrasound; and (5) not cooperating with the questionnaire survey.

### Collection of Clinical Data

All subjects underwent a comprehensive collection of general and clinical data, including age, sex, medical history, Epworth sleeping scale (ESS) evaluation, neurological examination, and head magnetic resonance imaging.

### Polysomnography

All the subjects were monitored for at least 8 h in the sleep center of our hospital using PSG (Compumedics, Australia). The PSG results were analyzed by professional sleep technicians, that had PSG technologist certification, by referring to the revised interpretation criteria of sleep stages and related events issued by the American Academy of Sleep Medicine version 2.1.

### Multiple Sleep Latency Tests

After PSG recording, the central electroencephalogram (C3-A2, C4-A1) and occipital electroencephalogram (O1-A2, O2-A1), left and right electrooculogram, electromyography, and electrocardiography were retained. The first nap was performed 2–3 h after the PSG examination. Afterward, 4–5 naps were taken at intervals of 2 h. Activities were avoided 15 min before each test. Sleep was avoided during the 2 h interval. A total of 15 min were recorded after falling asleep, including the 20 min before falling asleep. If only one SOREMP was recorded in the first four naps, an additional 5th nap was also recorded.

### Dynamic Cerebral Autoregulation Measurement

The participants were instructed to avoid alcohol, nicotine, and caffeine intake, and exercise for at least 12 h before the measurement. The measurement was performed in a quiet, dedicated research laboratory. First, the subjects had their baseline arterial blood pressure (Omron 711) and heart rates measured (15). Then beat-to-beat arterial blood pressure and continuous bilateral middle cerebral artery blood flow velocity were recorded for 10 min. The measurement data were then stored for further dynamic cerebral autoregulation examination analysis (16).

### Analysis of Dynamic Cerebral Autoregulation

Recorded data were processed using the MATLAB software (Math Works, Natick, MA, USA). The data analysis of dCA was performed using the transfer function analysis (TFA) (15). TFA between the arterial blood pressure and cerebral blood flow velocity was calculated as the quotient of the cross-spectrum of the two signals and the auto spectrum of arterial blood pressure in the low-frequency domain (0.06–0.12 Hz) to obtain the frequency-dependent estimates of phase difference (PD), where the derived parameters are considered to be the most relevant to autoregulation hemodynamics (17). A decreased PD represents impaired dCA. Coherence was calculated to estimate the reliability of the relationship between the two signals at the frequency domain, and the later statistical analysis was performed only if the coherence of the parameters was >0.5 (18).

### Statistical Analysis

The SPSS 23.0 software was used for statistical analysis. The Shapiro-Wilk test was used to assess the normal distribution of continuous variables. Measurement data with a normal distribution [including ESS, PD, age, mean arterial blood pressure (ABP), heart rate, and end-tidal CO_2_] were expressed as mean and standard deviation whereas data with skewed distribution were expressed as median (interquartile range). Categorical data (gender) were expressed as absolute values. Paired *t*-test was used to compare the differences between the two paired samples; ESS and PD in patients with NT1, before and after treatment. One-way ANOVA or Kruskal-Willis H test was used to compare the differences between the multiple groups of independent samples (ESS and PD of IH, NT1-RBD, NT1 + RBD, and control groups) based on the data distribution. Univariate and multivariate linear regression were used to assess the association of PD and clinical parameters including the total sleep time (TST), mean sleep latency in MSLT, sleep efficiency, percent of stage 1 non-REM (NREM) time in TST (stage N1), percent of stage 2 NREM time in TST (stage N2), percent of stage 3 NREM time in TST (stage N3), percent of REM sleep time in TST (REM sleep), and arousal index. In the *post-hoc* analysis, the Bonferroni method was used to calculate the adjusted *P*-value. *P*-values below 0.05 were considered statistically significant.

## Results

### Baseline Characteristics

In total, 48 participants were enrolled in this study, including 12 IH patients, 12 NT1-RBD patients, 12 NT1 + RBD patients, and 12 controls. There are no significant differences in gender, age, mean ABP, heart rate, and end-tidal CO_2_ among all the groups. The clinical characteristics of the participants are described in Table 1.

**Table 1:** Clinical characteristics in the patients with IH, NT1-RBD, NT1 + RBD, and controls.

	**Controls (*n* = 12)**	**IH (*n* = 12)**	**NT1-RBD (*n* = 12)**	**NT1 + RBD (*n* = 12)**	***P*-value**
Gender (male/female)	8/4	8/4	7/5	8/4	0.965
Age (years)	31.9 ± 7.9	39.8 ± 13.9	29.4 ± 10.3	34.9 ± 13.8	0.177
Mean ABP (mmHg)	83.3 ± 6.1	87.9 ± 5.0	84.7 ± 5.8	83.3 ± 5.3	0.061
Heart rate (bmp)	71.5 ± 6.2	70.9 ± 10.0	69.8 ± 7.7	70.2 ± 5.6	0.944
End-tidal CO_2_ (mmHg)	36.6 ± 3.6	36.7 ± 4.0	37.0 ± 3.9	36.6 ± 2.6	0.990

*IH, idiopathic hypersomnia; NT1-RBD, narcolepsy type 1 without rapid-eye-movement sleep behavior disorder; NT1 + RBD, narcolepsy type 1 with rapid-eye-movement sleep behavior disorder; ABP, arterial blood pressure*.

### ESS Score

**Figure 1:**
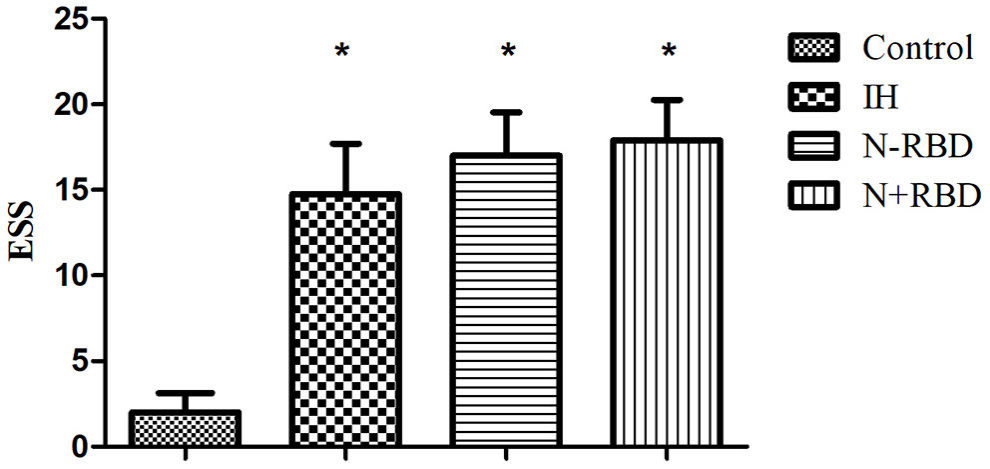
ESS scores in the patients with IH, NT1-RBD, NT1 + RBD, and controls. *Different from controls (*P* < 0.05). ESS, Epworth sleepiness scale.

ESS was used to assess the degree of sleepiness. The ESS scores of patients in the IH, NT1-RBD, and NT1 + RBD groups (14.8 ± 3.0, 17.0 ± 2.5, 17.9 ± 2.4) were higher than those of the control group (2.1 ± 1.1, *P* < 0.001) (Figure 1).

### dCA Parameters

**Figure 2:**
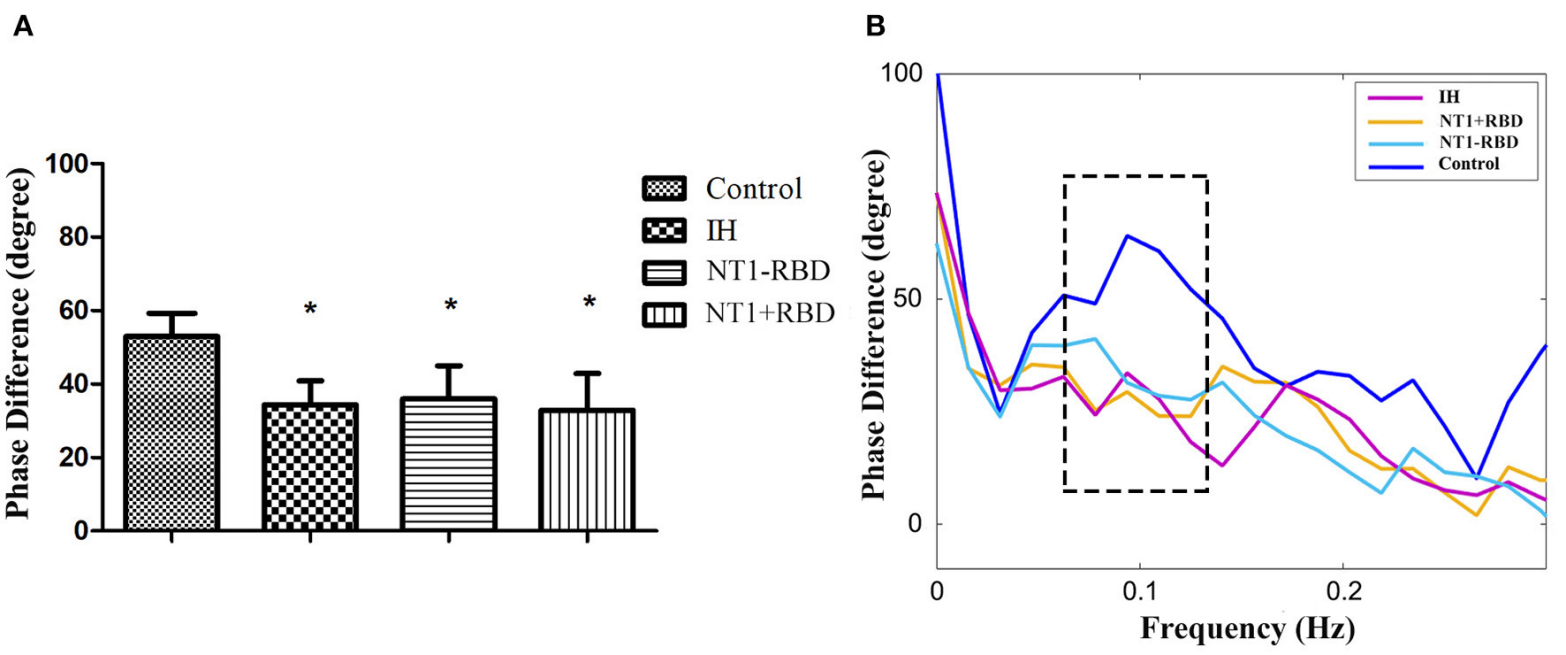
The autoregulatory parameter and statistical distributions in the patients with IH, NT1-RBD, NT1 + RBD, and controls. Statistical distributions of overall phase difference **(A)** and its transfer function **(B)** in each group. *Difference of overall phase difference in patients with IH, NT1-RBD, NT1 + RBD, and controls (*P* < 0.05).

The overall PD of patients in the IH, NT1-RBD, and NT1 + RBD groups (34.36 ± 6.51, 35.96 ± 8.99, 32.90 ± 9.98°) was significantly lower than that of the control group (52.98 ± 6.33°, *P* < 0.001) (Figure 2); however, there were no significant differences between the overall PD of the NT1-RBD and NT1 + RBD group (35.96 ± 8.99 vs. 32.90 ± 9.98°, *P* > 0.05).

### ESS and dCA Parameters of NT1 Before and After Treatment

**Figure 3:**
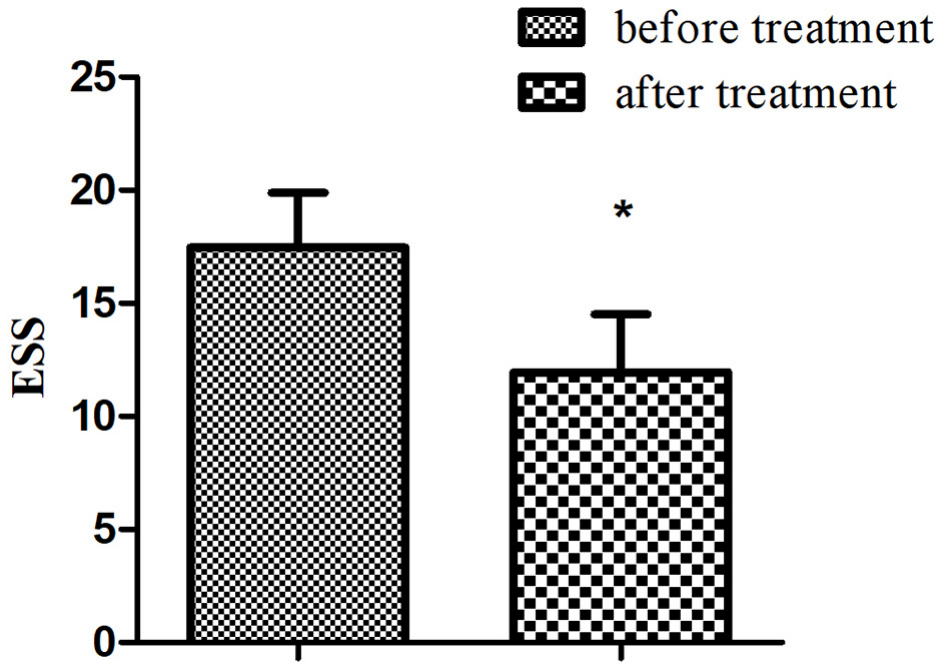
Comparison of ESS in narcolepsy type 1 group before and after treatment. *Represents statistical difference (*P* < 0.05).

**Figure 4:**
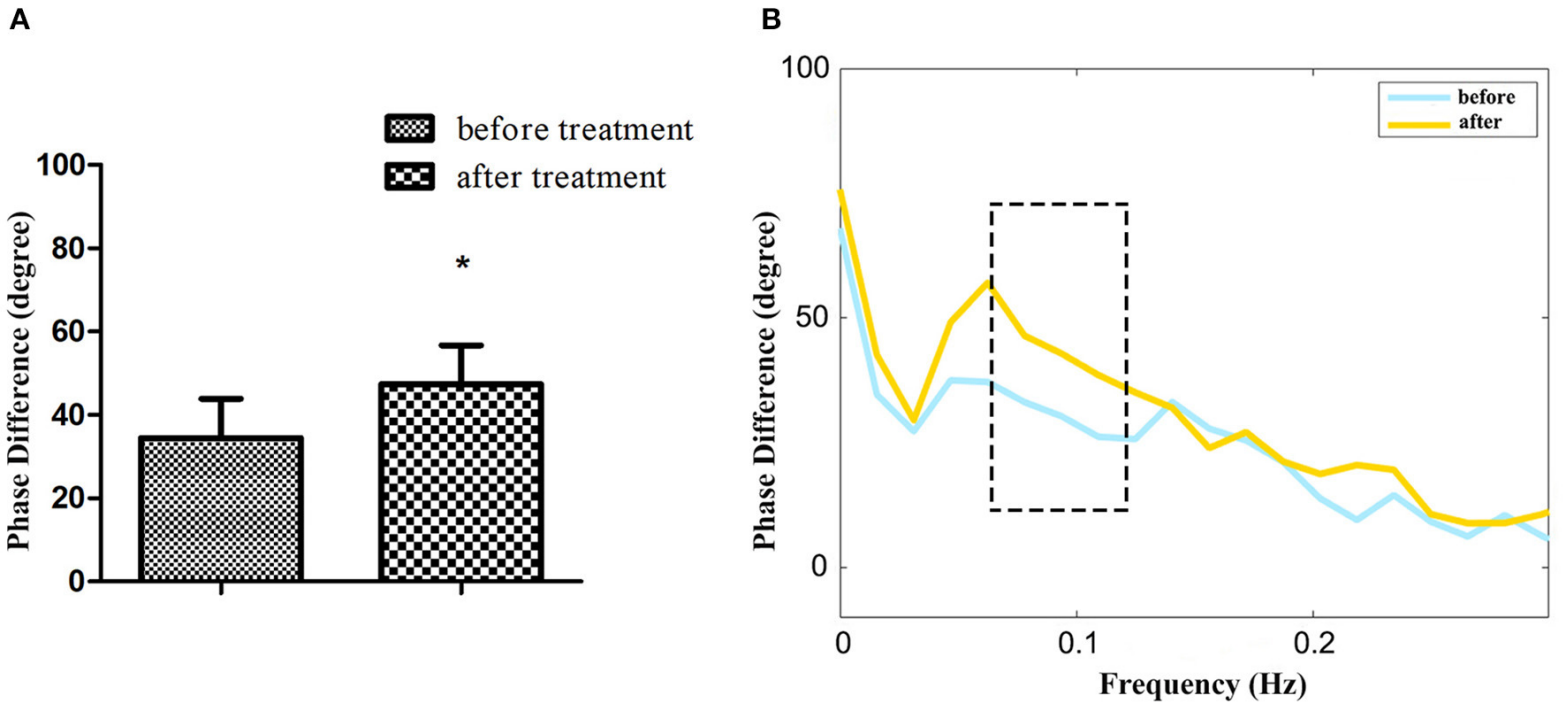
The autoregulatory parameter and statistical distributions in narcolepsy type 1 group before and after treatment. Statistical distributions of phase difference **(A)** and its transfer function **(B)** in narcolepsy type 1 group before and after treatment. *Represents statistical difference (*P* < 0.05).

Among 24 patients with NT1, the ESS and dCA were examined after 1-month treatment with methylphenidate (18 mg, once a day in the morning) and venlafaxine (75 mg, once a day in the morning). The ESS score decreased (11.96 ± 2.55 vs. 17.46 ± 2.43, *P* < 0.001) (Figure 3) and the overall PD increased after the treatment (47.37 ± 9.31 vs. 34.43 ± 9.42°, *P* < 0.001) (Figure 4).

### Univariable and Multivariable Analyses

The clinical parameters used in the univariable and multivariable analysis are shown in Table 2. In the univariable model, the overall PD correlated with mean sleep latency in MSLT (*P* < 0.001), sleep efficiency (*P* < 0.05), stage N1 (*P* < 0.05), and arousal index (*P* < 0.05). The multivariable model included mean sleep latency in MSLT, sleep efficiency, stage N1, stage N2, stage N3, and arousal index. Mean sleep latency in MSLT (*P* < 0.05) was an independent factor that influenced the overall PD (Table 2).

**Table 2:** Univariable and multivariable analysis of polysomnography parameters associated with the overall phase difference.

**Factors**	**Overall phase difference (°)**			
	**Univariable analysis**		**Multivariable analysis**	
	**β**	***P***	**β**	***P***
TST (min)	0.107	0.555		
Mean sleep latency in MSLT (min)	0.647	<0.001^a^^b^	0.470	0.016^b^^c^
Sleep efficiency (%)	0.416	0.016^a^^b^	0.059	0.785
Stage N1 (%)	−0.559	0.001^a^^b^	−0.515	0.128
Stage N2 (%)	0.330	0.060^a^	−0.168	0.511
Stage N3 (%)	0.298	0.092^a^	0.246	0.379
REM sleep (%)	0.238	0.183		
Arousal index	−0.376	0.031^a^^b^	−0.002	0.993

*TST, total sleep time; MSLT, multiple sleep latency test; REM, rapid eye movement; stage N1, percent of stage 1 non-REM (NREM) time in TST; stage N2, percent of stage 2 NREM time in TST; stage N3, percent of stage 3 NREM time in TST; REM sleep, percent of REM sleep time in TST*.

a *Nominally significant values (P <0.1) included in the multivariable model*.

b *P-value < 0.05 (statistically different)*.

c *Independent factor that influences overall phase difference*.

## Discussion

We found the cerebral autoregulatory parameter compromised in patients with central disorders of hypersomnolence. There were no significant differences in the degree of impairment between the NT1-RBD and NT1 + RBD patients. The ESS score and dCA improved after medication for the NT1 patients. The mean sleep latency in MSLT was independently associated with impaired dCA.

Studies have found abnormal CA in NT1 patients, evident by hypoperfusion of the hypothalamus, thalamus, prefrontal cortex, hippocampus, and cingulate gyri by single-photon emission computed tomography (2, 19), and abnormal CA is most likely implicated in the progression of neurological symptoms (such as cataplexy and sleep paralysis). The pathogenesis of compromised dCA in NT1 patients is the deficiency of hypocretin and monoaminergic neurons (9, 20). Hypocretin is involved in REM sleep and motor regulation through its effects on monoaminergic cells, including dopamine (DA), norepinephrine (NA), 5-hydroxytryptamine (5-HT), and other neurons (9, 21). NA is a sleep autonomic neuromodulating transmitter, which is an important factor for dCA (10). 5-HT is a vasoactive substance and has a potential role in dCA (11). In addition, hypocretin deficiency and a lower concentration of monoaminergic neurotransmitters lead to decreased activated projection to the basal forebrain and tuberomammillary nucleus; they are responsible for the maintenance of cortical arousal (22). Furthermore, the hypocretin neurons are located in the lateral hypothalamus and around the fornix, which projects to the regulatory centers of several autonomic nerves, including the autonomic neurons in the periaqueductal gray matter, the nucleus tractus solitarius, the nucleus ambiguous, the dorsal vagal nucleus, and the intermediolateral column of the spinal cord. Reduced or absent hypocretin levels in the inferior colliculus may reduce sympathetic excitability in NT1 patients (23). Our study showed that the dCA was impaired in NT1 patients, which may be related to hypocretin and monoamine neurotransmitter deficiency.

Narcolepsy is the second cause of RBD. Knudsen et al. found hypocretin deficiency to be independently associated with the prevalence of RBD outcomes (symptoms and muscle activity) during REM sleep in narcolepsy (24), which may explain the absence of a significant difference between the overall PD in the NT1-RBD and NT1 + RBD group; they may share the same mechanism. RBD sometimes can be the heralding symptom of NT1, forerunning the occurrence of cataplexy (25).

The pathological mechanism of IH is currently unclear. Studies have shown a normal concentration of hypocretin in the cerebrospinal fluid of IH patients; however, with low levels of DA, NA and 5-HT metabolites (8, 26, 27). In addition, IH patients have autonomic symptoms (cold extremities, palpitations, and fainting episodes) (27). We hypothesize that the impaired dCA in IH patients may be related to the lower concentration of monoaminergic neurotransmitters.

Drug therapy for narcolepsy in this study included methylphenidate and venlafaxine. Methylphenidate is a non-competitive DA reuptake blocker, which inhibits 5-HT and NA to a certain extent. Venlafaxine is a 5-HT-NA reuptake inhibitor that increases the concentration of 5-HT and NA. After 1-month treatment with the two drugs, the symptoms of EDS and the dCA improved in patients with narcolepsy, which may be related to the increased blood concentration of 5-HT and NA, and is consistent with previous reports (28, 29).

The MSLT is an objective test that measures the tendency to fall asleep under controlled conditions. It is based on the notion that sleep latency reflects the underlying physiological sleepiness (30). Since the mean sleep latency is shortened in IH and NT1 patients on MSLT, we found the mean sleep latency in MSLT to be independently associated with the impaired dCA. The potential mechanisms are still unclear. We hypothesize the 5-HT and NA levels to be a potential mechanism that explains the underlying relationship between the mean sleep latency and the dCA; larger sample size is required to confirm this result.

Our study shows that dCA is impaired in patients with central disorders of hypersomnolence, which may involve the monoaminergic mechanism; the relationship with hypocretin remains to be explored. The compromised dCA in patients with central disorders of hypersomnolence can explain and predict the clinical symptoms, and provide a new dynamic evaluating method for central disorders of hypersomnolence.

This study has some limitations. First, we did not examine the hypocretin of cerebrospinal fluid. Second, we did not detect the monoaminergic neurotransmitters in the participants' serum. Third, the sample size of this study is small, which may limit the analysis.

## Conclusion

The dCA is impaired in patients with central disorders of hypersomnolence. The dCA impairment occurs irrespective of NT1-RBD/+RBD. The ESS score and dCA improved in patients with NT1 after medication. The mean sleep latency in MSLT was independently associated with impaired dCA.

## Data Availability Statement

The raw data supporting the conclusions of this article will be made available by the authors, without undue reservation.

## Ethics Statement

The studies involving human participants were reviewed and approved by the Ethics Committee of the First Hospital of Jilin University. Written informed consent to participate in this study was provided by the participants' legal guardian/next of kin. Written informed consent was obtained from the individual(s), and minor(s)' legal guardian/next of kin, for the publication of any potentially identifiable images or data included in this article.

## Author Contributions

FD, YZ, and ZG wrote the manuscript. RZ conducted the data acquisition and data analysis. QT prepared the figures. YL analyzed the PSG. ZW and YY managed the study and edited the final manuscript. All authors contributed to the article and approved the submitted version.

## Conflict of Interest

The authors declare that the research was conducted in the absence of any commercial or financial relationships that could be construed as a potential conflict of interest.
